# Inferior Wall ST-elevation Myocardial Infarction Complicated by Ventricular Septal Defect and Free Wall Pseudoaneurysm with Rupture

**DOI:** 10.7759/cureus.3805

**Published:** 2018-12-31

**Authors:** Salma Khatoon, Michael Goyfman, Sepideh Nabatian, Sonia Henry, Steinberg Bart

**Affiliations:** 1 Internal Medicine, Long Island Jewish Forest Hills Hospital, New York, USA; 2 Cardiology, Long Island Jewish Forest Hills Hospital, New York, USA

**Keywords:** myocardial infarction, ventricular septal rupture, complications

## Abstract

Myocardial infarction (MI) is associated with complications in spite of appropriate management. The incidence of mechanical complications declined over time secondary to reperfusion therapies, improved control of blood pressure, the use of beta blockers and angiotensin-converting enzyme inhibitors, and aspirin. A high degree of suspicion is required, especially in elderly patients with complications post-PCI (percutaneous coronary intervention). Herein, we present a case of elderly male diagnosed with an inferior wall MI who had a PCI. He was found to have a post-infarction ventricular septal rupture (VSR) and basal inferior wall aneurysm that progressed over three weeks to a myocardial free wall rupture with hemopericardium. This case emphasizes the need for close monitoring of complications.

## Introduction

Acute myocardial infarction (MI) can be associated with pericardial complications, conduction abnormalities, and mechanical complications. Mechanical complications include rupture of the interventricular septum, rupture of the papillary muscle causing acute mitral regurgitation, as well as rupture of the left ventricular (LV) free wall. The incidence of free wall rupture was approximately 4% between 1977 and 2006 [1]. The incidence declined over time secondary to reperfusion therapies, improved control of blood pressure, the use of beta blockers and angiotensin-converting enzyme inhibitors, and aspirin. The frequency of ventricular septal rupture (VSR) has been reported to be about half that of free wall rupture [1]. On an average, free wall rupture occurs between three to five days after an MI, ranging from one to 14 days. Ventricular free wall rupture is an important but under-recognized cause of death after myocardial infarction. We present a case of post-infarction VSR and basal inferior wall aneurysm which progressed over three weeks to a myocardial free wall rupture with hemopericardium.

## Case presentation

**Figure 1:**
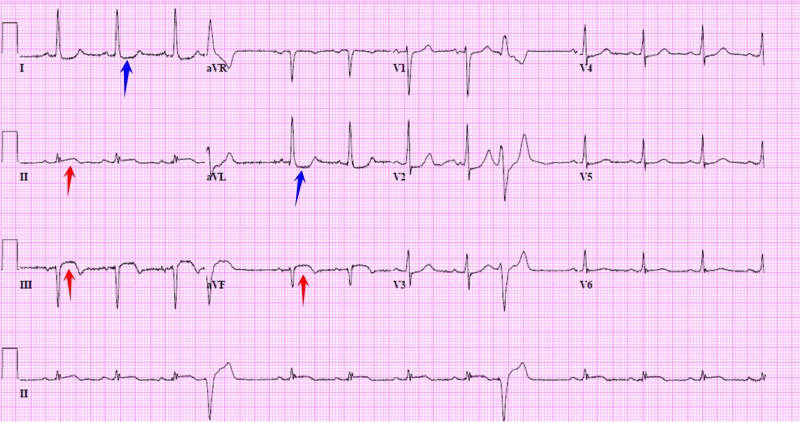
Electrocardiogram (EKG) upon admission EKG showing sinus rhythm with ST elevations in leads II, III, aVF (red arrows) and reciprocal ST depressions in leads I and aVL (blue arrows).

An 87-year-old male former smoker with hypertension, hyperlipidemia, and previous history of coronary artery disease (CAD) status-post percutaneous coronary intervention (PCI) 20 years ago, presented with intermittent chest discomfort for one week associated with lightheadedness. On admission, he was hypotensive with 70/54 mmHg and pulse rate of 69/min. Auscultation revealed no murmurs. An electrocardiogram (EKG) showed sinus rhythm with ST elevations in leads II, III, and aVF and reciprocal ST depressions in leads I and aVL (Figure 1).

**Figure 2:**
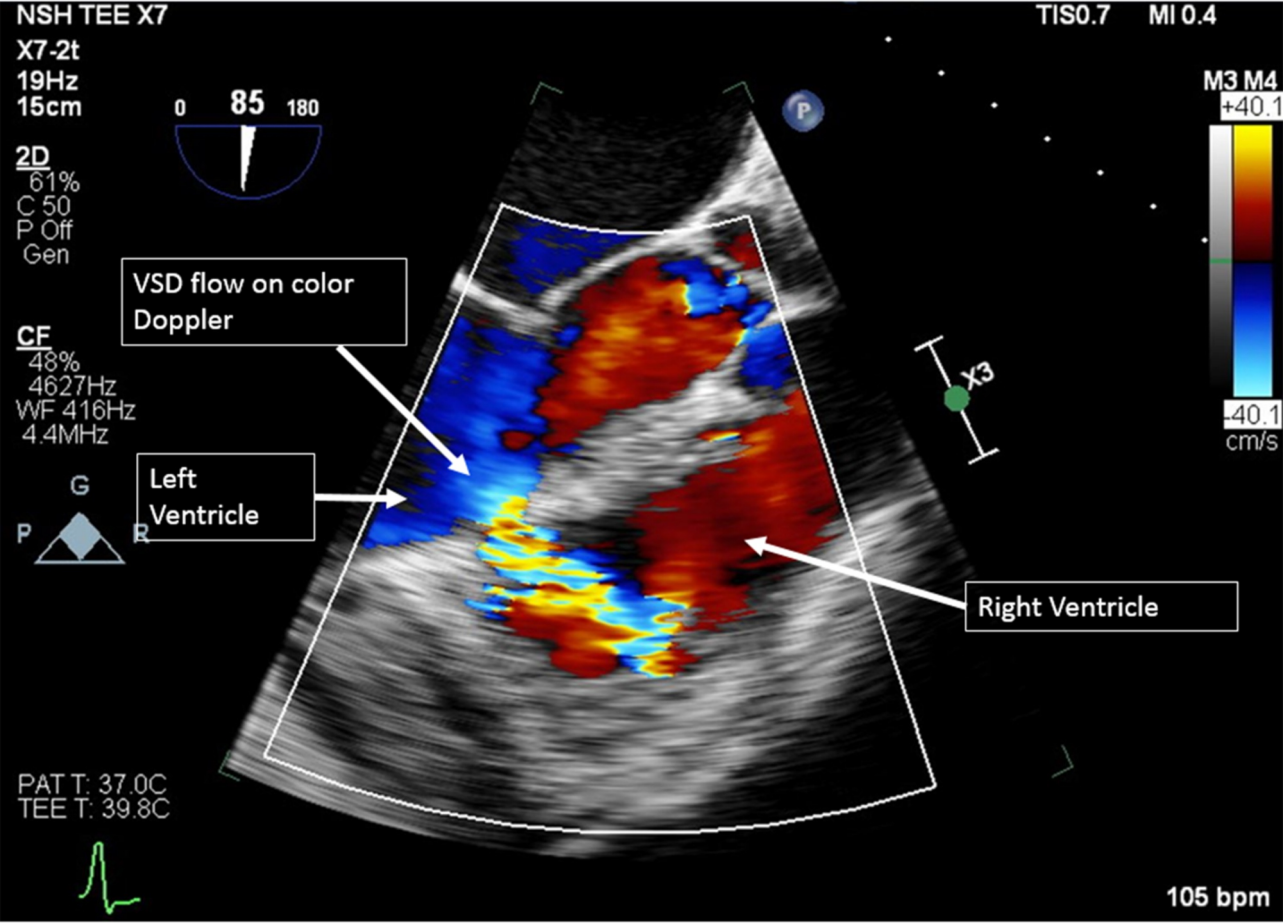
Transthoracic echocardiogram (TTE) showing VSD flow VSD: ventricular septal defect

The patient was treated with fluid boluses, aspirin 325 mg, a clopidogrel load of 600 mg, a heparin drip, and underwent urgent cardiac catheterization. Angiogram revealed a 99% stenosis in the right coronary artery (RCA) and 90% stenosis in the proximal left circumflex (LCx). A drug-eluting stent (DES) was placed in the RCA. An LCx intervention was staged the next day secondary to acute kidney injury. An echocardiogram performed on the day of admission showed mild LV systolic dysfunction with an ejection fraction (EF) of 55-60% and hypokinesis of the inferior and inferolateral wall. The patient’s nine-day hospital course was uneventful. Four days after discharge, he again presented to the ED with acute dyspnea, a new murmur, and congestive heart failure with hypoxia (O2 saturation 79% on room air), initially treated with oxygen and intravenous (IV) diuretics. A computed tomography angiogram (CTA) was negative for pulmonary embolus but was concerning for a ventricular septal defect (VSD). Echocardiogram revealed an LV basal inferior wall aneurysm with a VSD located at the inferior portion of the ventricular septum (Figure 2).

**Figure 3:**
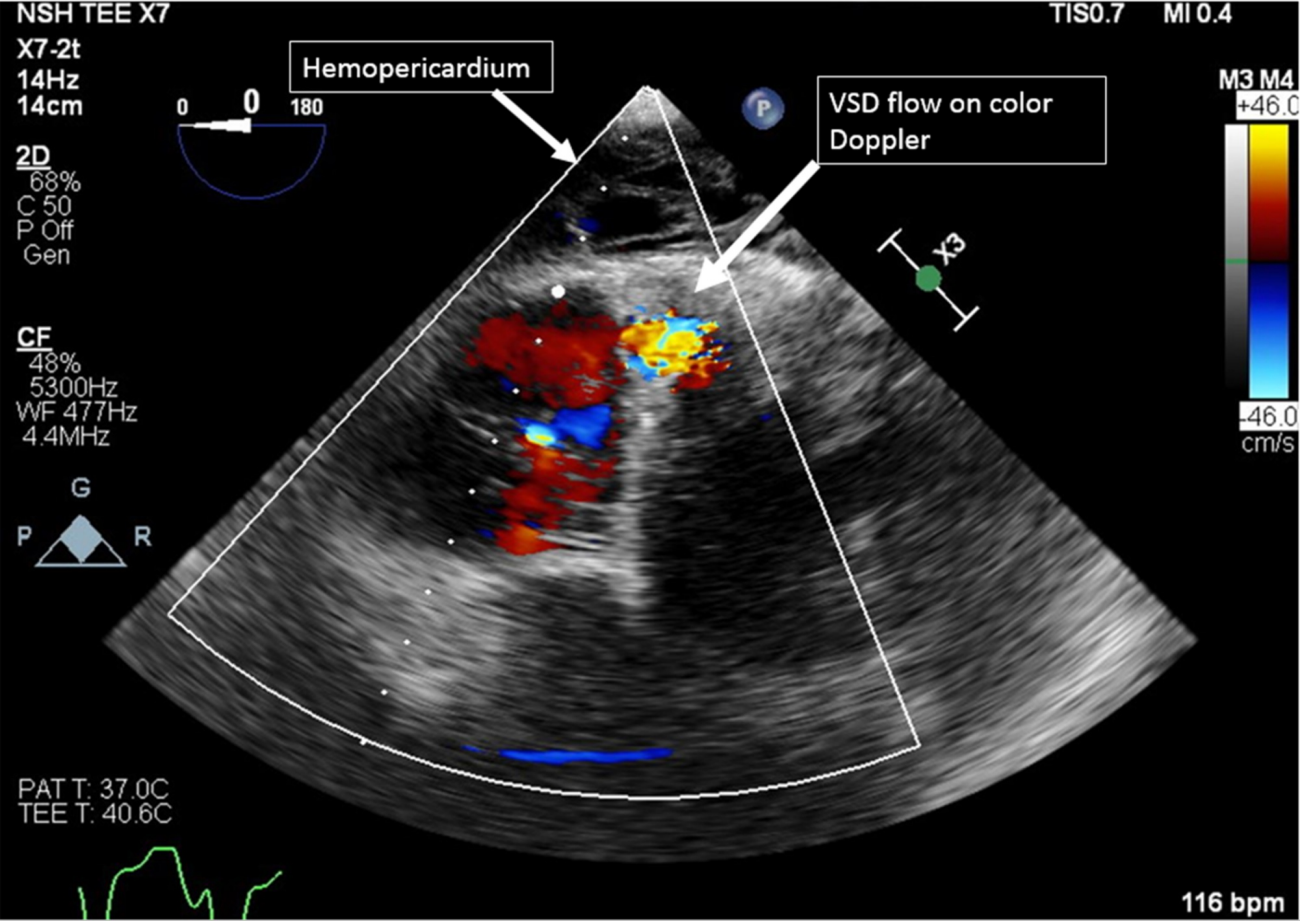
Transesophageal echocardiography (TEE) image of hemopericardium VSD: ventricular septal defect

Cardiac magnetic resonance imaging (MRI) confirmed a small defect within the inferior portion of the interventricular septum consistent with a post-MI VSD, measuring 8 x 11 mm. A percutaneous repair was planned as the surgical risk was deemed high. The patient’s hospital course was complicated by an upper gastrointestinal (GI) bleed and paroxysmal atrial fibrillation prior to attempting the procedure. The patient’s troponin-T was 0.15 ng/L on admission which later trended down to 0.07 ng/L. Once the patient was intubated for the percutaneous repair, intraprocedural transesophageal echocardiography (TEE) was performed, noting a walled-off myocardial free wall rupture with a large amount of clotted blood in the pericardium (Figure 3).

The VSD closure was aborted. The patient’s family opted for terminal extubation, and the patient died the next day.

## Discussion

Post-myocardial infarction VSR is a serious complication with high mortality rates, even with surgical intervention [2]. There are case reports of simultaneous left ventricular pseudoaneurysm and ruptured VSD following acute MI, whereas, with our case, the events appear sequential [3]. A pseudoaneurysm forms when cardiac rupture is contained by adherent pericardium or scar tissue [4-5]. These contain no endocardium or myocardium and should be diagnosed early due to the high risk of rupture. Pseudoaneurysms are seen primarily in the inferior or posterolateral wall after MI, as was the case with our patient. VSR is a well-recognized complication, and with the use of thrombolytic agents, the incidence has decreased from 1% to 2% in the pre-thrombolytic era to 0.2% currently [6-8]. In patients in the Global Utilization of Streptokinase and Tissue Plasminogen Activator for Occluded Coronary Arteries (GUSTO-I) trial, a ventricular septal rupture was suspected in 140 patients (0.34%) and confirmed by a retrospective review in 84 (0.2%) [9]. Thus, reperfusion therapy has decreased the incidence of septal rupture. Risk factors for VSD include hypertension, age > 60, and nonsmoking status. Angina or infarction may lead to myocardial preconditioning as well as to the development of coronary collaterals, both of which reduce the likelihood of septal rupture [10]. VSR should be suspected clinically after MI in the setting of a new murmur, chest pain, new onset heart failure, or cardiogenic shock. In patients with an inferior MI, septal ruptures involve the basal inferoposterior septum and are often complex with a serpiginous path. Ventricular septal rupture, regardless of being inferior or anterior, involves a right ventricular infarction [11]. The median time from the onset of symptoms of acute MI to rupture is generally 24 hours or less in patients who are receiving thrombolysis [12]. The incidence of ventricular free wall rupture is around 0.8 - 6.2% and thrombolytic therapy does not reduce risk as opposed to primary PCI. Rupture occurs typically between one and seven days after the infarction, and one-third of myocardial ruptures (pseudoaneurysms) are associated with VSR. Multimodal cardiac imaging using ventriculography, echocardiography, and cardiac MR provide information about the accurate and complete delineation of VSR. Medical management includes hemodynamic stabilization as a bridging therapy towards surgical repair or percutaneous intervention. Current guidelines recommend urgent VSD repair or closure irrespective of hemodynamic status [13-15]. Ultimately, the choice of intervention varies with the clinical presentation. Surgical repair has on overall 30-day mortality of 65% in one study, with a 74% mortality for posterior VSDs, in particular [16]. Transcatheter VSD closure has been a viable treatment option, involving sutures buttressed with Teflon felt. Other options include sutureless techniques using fibrin glue and collagen hemostats with a patch.

## Conclusions

This case demonstrates the evolution of a late presentation inferior MI progressing post-reperfusion ventricular septal rupture and inferior aneurysm with further progression to a free wall rupture. A high index of suspicion is to be emphasized, especially in elderly patients with complications post-PCI who may need early urgent closure of the septal rupture.
